# Efficacy of Connective Tissue with and without Periosteum in Regeneration of Intrabony Defects

**DOI:** 10.5681/joddd.2014.035

**Published:** 2014-12-03

**Authors:** Vahid Esfahanian, Hedayatollah Golestaneh, Omid Moghaddas, Mohammad Reza Ghafari

**Affiliations:** ^1^Assistant Professor, Department of Periodontics, Dental School, Islamic Azad University Isfahan (Khorasgan) Branch, Isfahan, Iran; ^2^Assistant Professor, Department of Periodontics, Dental School, Islamic Azad University, Tehran, Iran; ^3^Postgarduate Student, Department of Periodontics, Dental School, Islamic Azad University Isfahan (Khorasgan) Branch, Isfahan, Iran

**Keywords:** Connective tissue, guided tissue regeneration, periosteum, periodontal pocket

## Abstract

***Background and aims.*** Connective tissue grafts with and without periosteum is used in regenerative treatments of bone and has demonstrated successful outcomes in previous investigations. The aim of present study was to evaluate the effectiveness of connective tissue graft with and without periosteum in regeneration of intrabony defects.

***Materials and methods.*** In this single-blind randomized split-mouth clinical trial, 15 pairs of intrabony defects in 15 patients with moderate to advanced periodontitis were treated by periosteal connective tissue graft + ABBM (test group) or non-periosteal connective tissue graft + ABBM (control group). Probing pocket depth, clinical attachment level, free gingival margin position, bone crestal position, crest defect depth and defect depth to stent were measured at baseline and after six months by surgical re-entry. Data was analyzed by Student’s t-test and paired t-tests (α=0.05).

***Results.*** Changes in clinical parameters after 6 months in the test and control groups were as follows: mean of PPD reduction: 3.1±0.6 (P<0.0001); 2.5±1.0 mm (P<0.0001), CAL gain: 2.3±0.9 (P<0.0001); 2.2±1.0 mm (P<0.0001), bone fill: 2.2±0.7 mm (P<0.0001); 2.2±0.7 mm (P<0.0001), respectively. No significant differences in the position of free gingival margin were observed during 6 months compared to baseline in both groups.

***Conclusion.*** Combinations of periosteal connective tissue graft + ABBM and non-periosteal connective tissue graft + ABBM were similarly effective in treating intrabony defects without any favor for any group. Connective tissue and perio-steum can be equally effective in regeneration of intrabony defects.

## Introduction

Regeneration occurs in periodontology using several methods, including autogenous bone graft,^1^ bone substitutes,^2^ guided tissue regeneration^3^ or a combination of these methods.^4^ Guided tissue regeneration (GTR) uses either a resorbable or a non-resorbable barrier membrane to prevent the migration of epithelial cells, bone and gingival tissues to the wound area and will also provide an opportunity for accumulation of cells in periodontal fibers.^5-8^ However, based on the literature, there is no difference between resorbable and non-resorbable membranes in terms of treatment outcomes.^9-11^ Because of higher cost, need for a second surgery for membrane removal, complexity and bacterial accumulation of non-resorbable membranes, absorbable membranes are preferable.^12,13^

Bone grafts are used in the treatment of alveolar bone lesions because of their osteoconductive or osteoinductive properties and to maintain the space under the membrane and prevent it from collapsing into the bone defect.^14-17^ They also prevent the flap from collapsing and facilitate wound stability, which provides space to enable the regeneration process.^6^ One of these bone graft materials is bovine bone matrix material (Anorganic Bovine Bone Material—ABBM), which is a xenograft. This material has a high osteoconductive property and can bound with the bone.^18-20^ ABBM is extracted from natural bovine bone matrix and has been shown to be tolerable by the receiver site tissues, without causing allergic reactions.^21^ Use of ABBM alone or in combination with a resorbable membrane (collagen) or a non-resorbable membrane (expanded Polytetraflouroethylene—ePTFE) facilitates periodontal tissue regeneration in intra-osseous lesions.^22^ In addition, when the bone substitutes with bovine bone origin and collagenous membranes are used for periodontal regeneration, more attachment can be achieved in comparison with the flap surgery debridement.^17^ The periosteum, as a structure rich in osteoprogenitor cells, has been used with a regenerative potential.^23,24^ Having the ability to stimulate osteogenesis in the periodontally diseased area, periosteal grafts can be considered as a good alternative in regenerative modalities.^25^ Osteogenic progenitor cells available in the periosteum work with osteoblasts in initiating the cell-differentiation process of bone repair.^25^ In addition, the periosteum can act as a barrier rigid enough to maintain the space to allow essential cells such as osteoblasts to migrate in and regenerate lost periodontal tissues.

On the other hand, gingival connective tissue cells contain mesenchymal cells and have osteogenic, chondrogenic and osteoblastic capacity.^15,26-29^, These cells are also able to modulate the immune system.^30^ In fact, gingival tissue is a richer source of mesenchymal stem cells in comparison with bone marrow.^31^ Palatal autogenous connective tissue graft is regarded a proper treatment with advantages like lower cost, availability and adaptability.^26,27,32^ Perisoteal and non-periosteal connective tissue grafts have been used in regenerative treatments and both show successful results, but no available study has compared these two methods to date.^14,26-28,32,33^ Therefore, this study was performed to compare the effect of periosteum on palatal connective tissue grafts in association with ABBM on the clinical parameters in the treatment of vertical alveolar bone defects.

## Materials and Methods

This single-blind, randomized, split-mouth, controlled clinical trial was registered under the code IRCT2012101611133N1 in Iranian Registry of Clinical Trials. Fifteen patients with moderate to severe periodontitis that sought periodontal treatment at the Department of Periodontics, Dental School, Khorasgan (Isfahan) Branch, Islamic Azad University, were included in this study. Inclusion criteria consisted of sites with at least a 5-mm probing pocket depth after phase one therapy, a defect depth of at least 3 mm, a plaque index of 25% or less according to O’Leary plaque index^34^ before surgery, vital teeth or nonvital teeth with an appropriate root canal therapy and at least one pair of vertical defects in non-adjacent teeth. The patients were all informed about the study design and informed consents were signed. Patients with any systemic diseases, asthma and allergies, pregnant or breast feeder patients, patients with other types of chronic periodontitis or furcation involvement of grade 3 and 4, grade 2 or 3 tooth mobility, patients with a history of periodontal surgery six months before, patients using any medications during the previous three months and patients with abnormal platelet counts one month before surgery were excluded. To assess the oral health of the patients, simplified oral debris index (DI-S) was employed in the following manner: no debris or dye (score 0), presence of debris in one-third of the cervical portion (score 1), debris in more than two-thirds of the cervical portion of the (score 2), and debris in more than two-thirds of the tooth (score 3).^35,36^ Patients with DI-S of 0 and 1 around their teeth with intra-bony defects were included in this study.

Prior to the surgical phase, oral health instructions were presented, including how to properly brush using the Bass method, flossing, and use of an interdental toothbrush twice a day (morning and night). After oral health instructions, scaling and root planing was performed in two sessions by means of ultrasonic devices with a one-week interval. The patients were assessed at two-week intervals before the surgical phase. Other sites that required surgery underwent surgery before surgery for the present study.

After case selection based on the inclusion criteria, acrylic stents were made on a cast of each patient’s dental arch. To fabricate these acrylic stents, one third of the occlusal portion of the tooth with intra-osseous lesion and at least one tooth in the mesial and distal aspect of the tooth was covered with acrylic resin except in cases in which the tooth in question was the most distally positioned tooth in the arch.

Clinical parameters, including tooth position, probing pocket depth, clinical attachment level and gingival margin position, were recorded using this acrylic stent and a UNC-15 periodontal probe. To ensure perfect alignment of the acrylic index in place, the method of determining the distance to cementoenamel junction (CEJ) was used in the acrylic stent. Also for reproducible and reliable soft and hard tissue evaluations, guide slots were created in the stent. These tracks on preoperative casts were prepared so that the probe could be placed parallel with the long axis of the tooth. According to the entry angle of the probe, the groove slot was produced in the acrylic stent. This groove was a guide to determine the filling of lesions and record the changes in surgery.

For blinding the evaluations, all the measurements before and during surgery were performed by a post-graduate student under the supervision of a fully trained guide, who was unaware of the treatment type of each lesion. The patients were also unaware of the treatment type. The parameters measured were: probing pocket depth to stent (PPD-S), clinical attachment level to stent (CAL-S), free gingival margin to stent (FGM-S), crestal bone to stent (crest-s), defect depth to stent (Defect depth-S) and crestal bone to defect depth.

After soft tissue measurements prior to surgery, local anesthesia was provided with 2% lidocaine and 1:80,000 epinephrine (Darou Pakhsh, Tehran, Iran). Sulcular incision was made by scalpel blade No.15 in the mesial and distal aspects of the adjacent teeth on the buccal and lingual surfaces and a mucoperiosteal flap was elevated 3 mm beyond the margin of the lesion. After complete debridement of granulation tissues and removal of granulation tissue from the inner surface of the flap and the bony defect, the root surface was carefully planed. The acrylic stent was again placed and hard tissue parameters, including lesion depth, the distance between the alveolar crest the depth of the lesion and the distance between the alveolar crest and the acrylic stent, were measured by means of an UNC-15 periodontal probe. The number of the remaining walls of the lesion was also recorded. To retrieve palatal connective tissue graft, the palatal tissue thickness was measured after anesthesia with 2% lidocaine with 1:80,000 epinephrine to ensure the existence of at least 3 mm of thickness. A horizontal incision with a 3-mm distance from the palatal gingival margin was made by a scalpel blade No.15 at the site of the first molar to the first premolar, with 3 mm of surrounding bone and based on the length and width needed to cover the lesion. Two vertical incisions from the terminal points of the horizontal incision were made toward the midline of the palate. The mucosal flap was retracted by 3-0 silk suture (Supabon, Supa Medical Devices, Tehran, Iran). A thickness of 1‒1.5 mm of the underlying connective tissue was dissected by sharp dissection and was stored in normal saline-soaked gauze. The periosteum was not included in this thickness. On the other side of the palate, the connective tissue was removed in full thickness, including the periosteal tissue. In cases with thin palatal tissue and the need for sharp dissection, full-thickness tissue was harvested and the periosteum was excised with the blade outside the oral cavity. The palatal flap was sutured and covered with periodontal dressing. In both groups, ABBM (Geisttlich Pharma AG, Wolhusen, Switzerland) was used as the graft material. The granules were slightly formed by a sterile spatula. Connective tissue was shaped at each site to cover the defect without tension and in a manner to be secured on bony margins. Horizontal cross mattress sutures were used to stabilize the connective tissue graft in the desired position and the coronal edges of the flap were sutured using the 0-4 silk suture with the interrupted technique.

After surgery, 500-mg amoxicillin three times per day for a week, ibuprofen for pain control and 0.2% chlorhexidine mouth rinse twice per day for one month were prescribed. The patients were visited ten days later for suture removal and periodontal dressing was used for dressing the area again for another week. During the first month, the patients were visited every two weeks and each time the entire mouth was examined and professional prophylaxis was performed. After the first month the patients were visited monthly for six months after surgery. In all these visits, oral hygiene instructions were provided and debridement performed, if necessary.

Before re-entry surgery, the acrylic stent was placed by the same person and all of the soft tissue parameters were recorded. 2% lidocaine with 1:80,000 epinephrine was used for local anesthesia and sulcular incision was made by No. 15 blade on one tooth mesial and distal to the area. A mucoperiosteal flap was retracted. The acrylic index was placed again for hard tissue measurements. Subsequently, the amount of residual disease was treated accordingly. The flap was sutured with 0-4 silk sutures and periodontal dressing was placed. The patients were visited to remove the sutures and dressings after one week.

**Figure 1.**
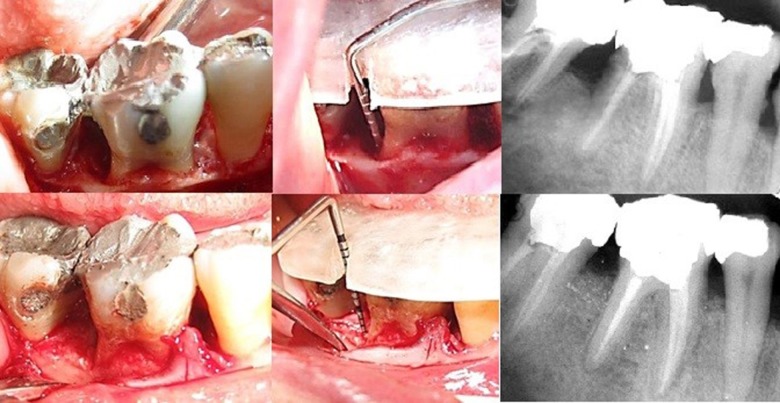
Comparison of clinical and radiographic appearances in defect resolution at baseline and six months after treatment in the test group (periosteal connective tissue graft + ABBM).

**Figure 2.**
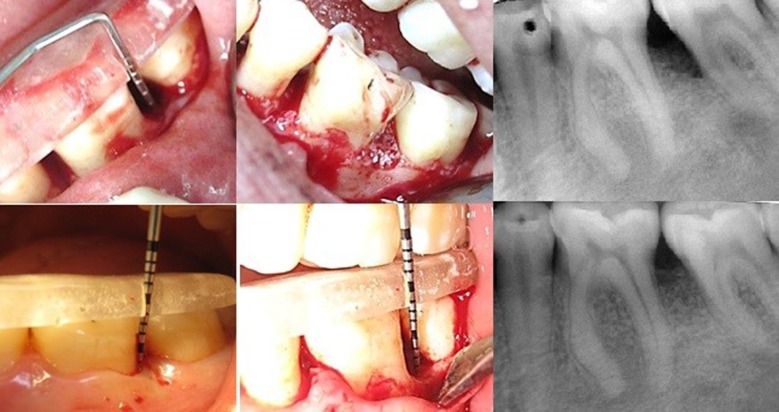
Comparison of clinical and radiographic appearances in defect resolution at baseline and six months after treatment in the control group (non-periosteal connective tissue graft + ABBM).

Figures 1 and 2 show the surgical steps and radiographs of the study groups. Data were analyzed by independent t test to compare the groups. In addition, to compare the results in each group, paired t-test (α=0.05) was used. P<0.05 was considered statistically significant.

## Results

**Figure 3:**
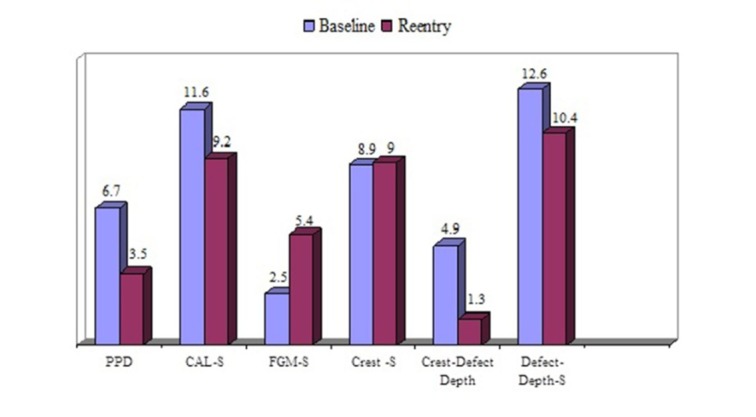
Mean differences in evaluated parameters in mm at baseline and after 6 months in the test group (periosteal connective tissue graft + ABBM).

**Figure 4.**
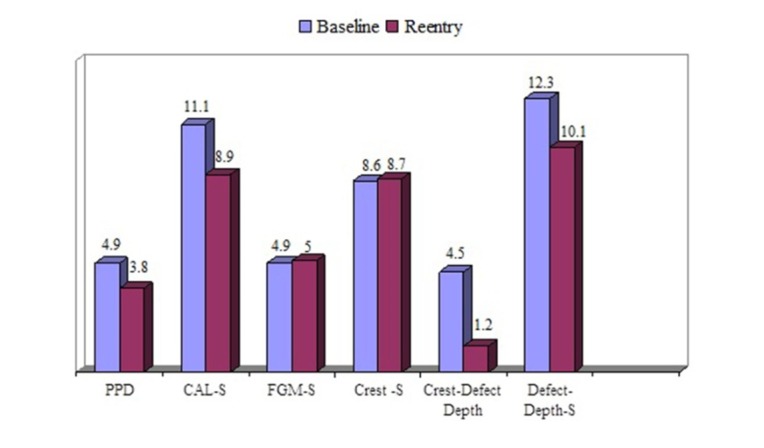
Mean differences in evaluated parameters in mm at baseline and after 6 months in control group (non-periosteal connective tissue graft + ABBM).

Fifteen patients, including seven men and eight women with a mean age of 48.2±5.8 years, were treated. Six thee-wall defects and nine two-wall defects were treated in the test group (connective tissue with periosteum), and four three-wall defects plus eleven two-wall defects were treated in the control group (connective tissue without periosteum). The results of the study are summarized in Table 1 and Figures 3 and 4.

**Table 1:** Mean changes in evaluated parameters in millimeters at baseline and after 6 months in test and control groups

Clinical parameters	Test group (periosteal connective tissue + ABBM)				Control group (non-periosteal connective tissue + ABBM)			
	Baseline	6 Months	P-value	Changes	Baseline	6 Months	P-value	Changes
PPD^1^	6.7±0.6	3.5±0.4	P<0.0001	3.1±0.6	6.3±0.5	3.8±0.7	P<0.0001	2.5±1.0
CAL^2^	11.6±0.8	9.2±0.5	P<0.0001	2.3±0.9	11.1±0.9	8.9±0.6	P<0.0001	2.2±1.0
FGM-S^3^	5.2±0.6	5.4±0.5	P=0.08	0.2±0.4	4.9±0.5	5.0±0.7	P=0.1	0.1±0.3
Crest-S^4^	8.9±0.6	9.0±0.4	P=0.84	0.03±0.6	8.6±0.5	8.7±0.5	P=0.69	0.07±0.6
Crest-Defect Depth	4.9±0.6	1.3±0.4	P<0.0001	3.6±0.6	4.5±0.6	1.2±0.5	P<0.0001	3.3±0.6
Defect Depth-S^5^	12.6±0.7	10.4±0.4	P<0.0001	2.2±0.7	12.3±0.6	10.1±0.6	P<0.0001	2.2±0.7
^1^Probing Pocket Depth (changes show depth reduction)								
^2^Clinical Attachment Level (changes show clinical attachment gain)								
^3^Free Gingival Margin to Acrylic Stent (changes show gingival recession)								
^4^Alveolar Bone Crest to Acrylic Stent (indicates crestal recession)								
^5^Defect Depth to Acrylic Stent (changes show defect fill)								
P<0.05 was considered statistically significant								

PPD-S reduction, CAL-S gain and bone fill significantly improved in the re-entry evaluations in both groups compared to baseline (P<0.0001). There were no significant changes in the distances between the free gingival margin and the alveolar bone crest to the stent in both groups (P values are shown in Table 1). The differences between the groups did not reach a statistically significant level.

## Discussion

It was initially thought that palatal connective tissue graft may proliferate into the defect site, but it was recently confirmed that this tissue can act as a tolerated biological barrier membrane. It prevents the epithelial cells from proliferation into the lesion site and no proliferation of a tissue graft of this type into the lesion is observed.^30^ Some studies have shown that the periosteal tissue can be used as a membrane in regenerative therapy, mainly because (1) this membrane is available, (2) there is no need for a second removal surgery, (3) no risk of transmission of diseases is observed, and (4) the outcome does not pose any hazards if exposure to the oral environment happens.^15,26,37^The periosteum, as a structure rich in osteoprogenitor cells, has been used with regenerative potential.^23,24^ Periosteal grafts have the capacity to stimulate osteogenesis in the periodontally diseased area and can be considered a good alternative in regenerative modalities.^25^ Osteogenic progenitor cells of the periosteum collaborate with osteoblasts, finally resulting in the differentiation of cells during bone repair.

On the other hand, it has been reported in literatures that gingival connective tissue itself contains mesenchymal cells even in higher levels of what can be found in bone marrow, conferring even superior osteogenic, chondrogenic and osteoblastic properties to the connective tissue.^30,31^ In several studies by Paolantonio et al^33^ and Moghaddas et al,^15,38,39^ comparable results were shown in utilizing palatal connective tissue graft without periosteum and collagenous membranes. Based on the results of the present study, the mean pocket depth reductions in the test group (periosteal connective tissue + ABBM) and control group (non-periostealconnective tissue + ABBM) were 3.1 mm and 2.5 mm, respectively. These measurements were reported as 2.2 mm by Kwan et al^26^ using periosteal connective tissue, and 2.6 mm by Moghaddas et al^15^ using connective tissue without periosteum. In addition, Moghaddas and Zamani^27^ found 3.5 mm of probing pocket reduction using palatal connective tissue graft alone, which is approximately in the range of the present study. Minor differences in the results might be attributed to differences in the details and the type of the treatments.

Based on the current results, attachment gain values in the test and control groups were 2.3 mm and 2.2 mm, respectively, indicating the effectiveness of both methods. These measurements were reported as 1.5 mm by Moghaddas et al,^15^ 2.1 mm by Moghaddas and Zamani,^27^ 3.2 mm by Moghaddas and Ghasemi,^14^ 2.8 mm by Moghaddas and Jalali,^38^ and 2.2 mm by Kwan et al.^26^ These values all fall within a narrow range.

Other studies, on the other hand, show that the treatment of bony defects with palatal connective tissue grafts can significantly reduce probing pocket depth and clinical attachment level gain in comparison with open flap debridement procedures.^33,40^ It has also been shown that palatal connective tissue grafts in combination with ABBM can significantly reduce probing pocket depth and improve clinical attachment gain.^16,39,41-43^, In these studies, reductions in probing pocket depths were 2.8 mm,^16^ 1.89 mm,^41^ 5.3 mm,^42^ and 3.7 mm,^43^ and clinical attachment level gains were 2.7 mm,^16^ 2.1 mm,^41^ 4 mm,^42^ and 3.3 mm.^43^

The results of the present study showed almost no change in the position of the gingival margin (gingival recession) in the test (periosteal connective tissue + ABBM; 0.2 mm, P=0.08) and control (non-periosteal connective tissue + ABBM; 0.1 mm, P=0.1) groups. The obtained values are comparable to those in studies by Moghaddas and Zamani^15^ (0.9 mm), Moghaddas and Ghasemi^14^ (0.5 mm) and Kwan et al^26^(0.3 mm). The minimal amount of gingival recession in the test and control groups is a great advantage of the two evaluated methods, since one reason for using regenerative methods is esthetic considerations. Preventing gingival recession, especially in the anterior region, provides a higher patient satisfaction. In studies that have compared collagen membranes and connective tissue in regeneration of intrabony defects, gingival margin positions have had significantly lower rates of recession with connective tissue, which is probably the result of simultaneous soft tissue augmentation provided by the connective tissue.^15,33,39^

Minor differences between the present results and those of previous studies^8,44^ can be justified by initial defect depth (the deeper the defect, the more gain in attachment and bone fill) and the differences in the type of the materials used. Previous researches have also emphasized the importance of minimal soft tissue manipulation techniques at the crestal area in preventing further soft tissue recession, which can lead to more attachment gain.^8,45^ The results of the present study indicate that palatal connective tissue can prevent recession. There were no significant differences between the two groups in terms of gingival recession after treatment. Previous research on comparison of collagen and palatal connective tissue membranes have shown no significant differences regarding treatment efficacy.^15,33,46^

In this study, bone fill in both groups was 2.2 mm. In addition, the defect resolutions comprising crestal bone resorption of almost 0.1 mm (0.03 mm in the test and 0.07 mm in the control group) was 2.2‒2.3 in both groups. A study by Moghaddas et al^15^ showed a 3.4-mm filling of the defect using the connective tissue + Bio-Oss+ Collagen, and Esfahanian et al^16^ observed a 2.6-mm defect fill. In another study by Moghaddas et al,^39^ filling of the defects was 2.3 mm. Paolantonio et al^33^ evaluated the treatment of vertical bone defects by open flap debridement, open flap debridement with GTR (collagenous membrane) and open flap debridement with periosteal connective tissue graft plus autogenous bone and showed that the filling of the lesions in the periosteal connective tissue group was more than the collagenous group (3.1 to 2.4). The researchers explained that using autogenous bone improves the results. It should be noted that in the mentioned study, despite the use of autogenous bone granules in conjunction with connective tissue, no graft material was used.

In a systematic review by Sculean et al,^47^ it was reported that the results improved by using a combination of membrane and graft material compared to the membrane alone. In the present study, no significant differences were found between “periosteal connective tissue + ABBM” and “non-periosteal connective tissue + ABBM” groups in terms of defect filling. It seems that using autogenous bone grafts beneath connective tissue in the bone defect is the reason for more filling in studies by Paolantonio et al^33^ and Femminella et al^48^ and it can be due to the osteogenic effects of autogenous bone graft material and the fact that it prevents the membrane from collapsing.

Kwan et al^26^ reported that periosteal connective tissue as a membrane shows better results in terms of clinical attachment gain (2.3 mm) and defect fill (2.6 mm) compared to the control group (open flap debridement alone). The researchers reported that the periosteal tissue can be used as a membrane for guided tissue regeneration. Also, higher rate of defect fill in the experimental group was attributed to the osteogenic property of the periosteum. In the present study, no differences were achieved utilizing periosteal and non-periosteal connective tissues. This may indicate the connective tissue itself has the ability to promote the regeneration process, similar to cases in which the periosteum is present. An important consideration is the fact that the periosteum which has an osteogenic capacity is the one harvested from a healthy site not the one from the periodontal flaps. That is why the periosteum in this study, similar to other studies, was harvested from the palate.

## Conclusion

Both the “periosteal connective tissue + ABBM” and “non-periosteal connective tissue + ABBM” treatments significantly improved the clinical parameters after six months, with no significant differences between the two groups. Thus, ABBM and palatal connective tissue with and without periosteum can be equally effective in intrabony defect regeneration with no superiority over each other.

## Aknowledgement

This article is based on a thesis submitted to the Dental School, Islamic Azad University Isfahan (Khorasgan) Branch, in partial fulfillment of the requirements for the Degree of MSc in dentistry (periodontics) and was financially self-supported.
